# *Ocimum basilicum*-Mediated Synthesis of Silver Nanoparticles Induces Innate Immune Responses against Cucumber Mosaic Virus in Squash

**DOI:** 10.3390/plants11202707

**Published:** 2022-10-13

**Authors:** Ahmed Abdelkhalek, Hamada El-Gendi, Fatimah O. Alotibi, Abdulaziz A. Al-Askar, Toufic Elbeaino, Said I. Behiry, Kamel A. Abd-Elsalam, Hassan Moawad

**Affiliations:** 1 Plant Protection and Biomolecular Diagnosis Department, ALCRI, City of Scientific Research and Technological Applications, New Borg El Arab City 21934, Egypt; 2 Bioprocess Development Department, Genetic Engineering and Biotechnology Research Institute, City of Scientific Research and Technological Applications, New Borg El-Arab City 21934, Egypt; 3 Department of Botany and Microbiology, College of Science, King Saud University, P.O. Box 2455, Riyadh 11451, Saudi Arabia; 4 Istituto Agronomico Mediterraneo di Bari (CIHEAM-IAMB), Via Ceglie 9, Valenzano, 70010 Bari, Italy; 5 Agricultural Botany Department, Faculty of Agriculture (Saba Basha), Alexandria University, Alexandria 21531, Egypt; 6 Plant Pathology Research Institute, Agricultural Research Centre, Giza 12619, Egypt; 7 Agriculture Microbiology Department, National Research Centre, Cairo 12622, Egypt

**Keywords:** silver nanoparticles, CMV, basil, squash, defense-related enzymes, gene expression

## Abstract

Cucumber mosaic virus (CMV) causes a significant threat to crop output sustainability and human nutrition worldwide, since it is one of the most prevalent plant viruses infecting most kinds of plants. Nowadays, different types of nanomaterials are applied as a control agent against different phytopathogens. However, their effects against viral infections are still limited. In the current study, the antiviral activities of the biosynthesized silver nanoparticles (Ag-NPs) mediated by aqueous extract of *Ocimum basilicum* against cucumber mosaic virus in squash (*Cucurbita* pepo L.) were investigated. The prepared Ag-NPs were characterized using scanning electron microscopy (SEM), dynamic light scattering (DLS), transmission electron microscopy (TEM), energy-dispersive X-ray spectroscopy (EDX), Fourier transform infrared spectroscopy (FTIR) and zeta potential distribution techniques. DLS, SEM, and TEM analyses showed that the Ag-NPs were spherical, with sizes ranging from 26.3 to 83 nm with an average particle size of about 32.6 nm. FTIR identified different functional groups responsible for the capping and stability of Ag-NPs. The zeta potential was reported as being −11.1 mV. Under greenhouse conditions, foliar sprays of Ag-NPs (100 µg/mL) promoted growth, delayed disease symptom development, and significantly reduced CMV accumulation levels of treated plants compared to non-treated plants. Treatment with Ag-NPs 24 h before or after CMV infection reduced CMV accumulation levels by 92% and 86%, respectively. There was also a significant increase in total soluble carbohydrates, free radical scavenging activity, antioxidant enzymes (PPO, SOD, and POX), as well as total phenolic and flavonoid content. Furthermore, systemic resistance was induced by significantly increasing the expression levels of pathogenesis-related genes (PR-1 and PR-5) and polyphenolic pathway genes (HCT and CHI). These findings suggest that Ag-NPs produced by *O. basilicum* could be used as an elicitor agent and as a control agent in the induction and management of plant viral infections.

## 1. Introduction

Continuous crop losses attributed to plant infection represent a pressing human challenge for food security [1]. Among them, plant viral infections account for considerable crop losses that may have reached 100% in some cases [2,3]. Cucumber mosaic virus (CMV) is among the most viral plant pathogens with the broadest range of hosts. The CMV is a multi-copy linear plus-sense RNA virus belonging to the family *Bromovirida.* The CMV infection could affect many cultivated plants, including tomato, melon, and pepper, due to its high incidence of crop losses in more than 1200 plant species [4]. Furthermore, several studies have shown that the virus can infect various wild plants [5,6]. This broad range of plant hosts, combined with the CMV’s significant ability to spread, adds more challenges to the viral control process. Several reasons explain the wide spread of CMV infection, such as the broad nonpersistent transmission through more than 80 aphid species through concise contact with infected plants, in addition to the mechanical transmission through contacting the infected and healthy plants through agriculture tools and labor [7,8].

CMV infection has resulted in severe mosaic and deformation of produced fruits, consequently affecting the squash crop’s production and marketing [9]. Squash plants (*Cucurbita pepo* L.) are among the CMV’s plant hosts. The plant fruits are cultivated worldwide for human consumption, especially in the Mediterranean territories and Europe [10,11]. The plant fruits’ high nutritional value encourages their consumption due to their high content of several essential vitamins (vitamins A, C, and B), minerals such as K+, and phenolic compounds. The low glycemic index of squash fruits indicates their consumption benefits, especially in diabetic and overweight conditions. Furthermore, several studies have reported the potential biological activities of various squash plant parts, including anticancer, antioxidant, and anti-diabetic potential [11,12]. 

Many efforts have been made to limit CMV infection, such as editing and breeding more resistant plant species [13,14], optimizing culturing processes to remove grasses, and using an insecticidal chemical to slow viral transmission. The capacity of CMV to circumvent plant resistance, as well as the high cost and environmental impact of excessive pesticide treatment, continue to be the key barriers to viral control [15,16]. The ineffective results for the currently used CMV control measures, in terms of expense and environmental impact, highlight the urgent need for new and effective eco-friendly control procedures. With respect to their broad promising biological activities, including medical, environmental, and industrial applications, there has recently been a growing interest in implementing NPs in agriculture [17,18]. Several studies reported different NPs’ ability to enhance plant resistance to various abiotic and biotic stresses [19,20,21]. Under viral challenge, NPs application was reported to enhance the plant systemic acquired resistance (SAR) system and several antioxidant mechanisms to alleviate infection and disease severity [22,23].

Several methods for preparing NPs have been presented. However, as environmental concerns develop, the green synthesis method emerges as a more environmentally friendly strategy that may reduce the environmental toxicity of NPs generated using standard physical and chemical methods [24,25]. Ag-NPs were extensively studied due to their wide range of antibacterial, antifungal, antiviral, and anticancer activities [17,26,27]. In plant pathology, Ag-NPs demonstrated antifungal and antiviral activity, as well as considerable nematicidal activity [28,29], improving plant growth indices and fruit output [30]. The application of Ag-NPs improved plant tolerance to a variety of biotic stressors, including phytopathogenic fungi [26,31] and worms [19,28]. Their application to plant viral diseases, however, is relatively limited [17,32]. As a result of the promising activity of Ag-NPs, the current study attempted eco-friendly production of Ag-NPs via green synthesis, using an extract from *Ocimum basilicum*. Ag-NPs were characterized by various techniques, including SEM, DLS, TEM, EDX, FTIR, and zeta potential distribution methods. The antiviral activity and effectiveness of the biosynthesized Ag-NPs in inducing SAR against CMV, as well as their effects on plant growth parameters, the level of CMV accumulation, antioxidant enzymes, and the transcriptional levels of defense-related genes, were also studied.

## 2. Materials and Methods

### 2.1. Plant Material and CMV Isolate

The CMV isolate (accession number OL348189) applied during the current study was previously isolated from CMV-infected squash plants [9]. The purified CMV isolate was used as a source of viral inoculum for all CMV-challenged treatments. The squash (*Cucurbita pepo* L.) cultivar NSH5 seeds used in the experiment were virus-free seeds provided by the Agriculture Research Center, Giza, Egypt.

### 2.2. Green Synthesis of Silver Nanoparticles (Ag-NPs)

The Ag-NPs were prepared through a green-ecofriendly reduction approach using *Ocimum basilicum* plant extract adapted from [19]. First, the plant leaves were washed several times with double distilled water (ddw) to remove any impurities, then air-dried and blended. The plant extract solution was prepared by homogenizing 10 g of plant powder into 100 mL of ddw for 120 min at 50 °C. To remove the remaining plant material, the solution was aseptically filtered through Whatman filter no. 1. After that, 10 mL of the plant extract was added to 90 mL of freshly prepared 1 mM AgNO_3_ solution (Sigma Aldrich, St. Louis, MO, USA) with mixing under dark conditions. The development of reddish-brown indicates the Ag++ reduction and formation of Ag-NPs. The reaction mixture was then centrifuged at 6000 rpm for 10 min, where the resulting precipitate was washed several times with ddw and one time with absolute ethanol. The precipitate was then dried at 50 °C for 24 h and used as a source for Ag-NPs in the following experiments. 

### 2.3. Characterization of the Green Synthesized Ag-NPs

The green synthesized Ag-NPs were characterized through several different instrumental approaches. The surface and morphological structures of the prepared Ag-NPs were elucidated through SEM using the JSM-6360 LA microscope (JEOL, Tokyo, Japan) and TEM using the JEM-2100 microscope (JEOL, Tokyo, Japan). The functional groups in the green synthesized Ag-NPs were evaluated using Fourier transform infrared spectroscopy (FTIR) using FTIR-8400S (SHIMADZU, Kyoto, Japan) according to the KBr disc method. A particle size analyzer confirmed the particle-size distribution (PSD) (MALVERN, ZETASIZER Ver.6.20, Malvern, UK).

### 2.4. The Experimental Greenhouse Design

The efficacy of Ag-NPs in alleviating the CMV infection in squash was evaluated under greenhouse conditions in pot experiments. The plastic pots (30 D × 28.6 W × 35.6 H cm) encompass a pre-sterilized soil mixture of clay, sand, and peat moss prepared in a ratio of 1:1:1. The experiment was allocated into four groups; the first group was for uninfected plants (Mock plant); the second group was the CMV-infected non-treated plants; the third group was the CMV-infected group that was prophylactically treated with Ag-NPs 24 h before CMV inoculation; and the final group represented the CMV-infected group that was treated with Ag-NPs 24 h after CMV inoculation. Each group was represented by five pots, where each pot comprised three plants, and all pots were growing in the greenhouse conditions of 28 °C/16 °C (day/night) with 70% relative humidity. The CMV-challenged treatments were carried out after two weeks of seeds sowing, according to Hafez et al. [33]. Briefly, carborundum (600 mesh) was dusted upon the true upper leaves of each plant. Then the leaves were mechanically inoculated with 20 µg/mL of CMV inoculum (prepared in 10 mM phosphate buffer (pH 7.2) and 0.1 sodium sulfite). In the treatment groups, the Ag-NPs solution (100 µg/mL) was foliar sprayed in all plants until the leaves were fully soaked at 24 h before CMV infection in group 3 (Pre-CMV) and 24 h after infection in group 4 (Post-CMV). After viral infection, all plant pots were cultivated for about three weeks under insect-proof greenhouse conditions and were observed daily for symptom development. Squash plants were collected 18 days after CMV infection (dpi) and analyzed for their shoot and root development. The harvested plants from each group were washed several times with running water. For subsequent analysis, two upper leaves per plant were collected from each pot (6 leaves/pot) and represented as a biological sample within the same treatment group (a total of 5 biological samples). Each biological sample was run in three technical replicates.

### 2.5. Evaluation of the Free Radical Scavenging Activity

The impact of Ag-NPs applications upon free radical scavenging activity was evaluated, as described previously [34]. Briefly, 2 mL of DPPH (2,2-Diphenyl-1-picrylhydrazyl, 0.05 M in methanol) was added to 100 µL of plant extract prepared in phosphate buffer pH 7.0 and incubated at 25 °C for 30 min. The reduction in the reaction absorbance was measured at 517 nm and expressed as a percentage (%) using the original DPPH absorbance as 100%. 

### 2.6. Total Phenolic and Flavonoid Contents Evaluation

The total phenolic compounds were evaluated through the Folin–Ciocalteau approach [35], where 2 mL of Folin–Ciocalteau reagent was added to 400 µL of the methanol extracted plant extract (0.5 g of dried plant: 25 mL of methanol 80%). After 5 min of incubation at room temperature, a solution of 7.5% Na_2_CO_3_ (*w*/*v*) was added to a final volume of 5 mL. The reaction mixture was vigorously shaken and incubated for 60 min in dark conditions. The developed color was measured at 750 nm where the total phenolic contents were determined using a standard curve of gallic acid. The aluminium chloride method was applied [36] with some modifications for total flavonoid contents evaluation. Briefly, plant extract in sodium phosphate buffer (pH 7.0, 500 µL) was mixed with 10% of aluminium chloride (100 µL), 1 M of potassium acetate (100 µL), and 1500 µL of methanol to a final reaction volume of 5 mL. After 30 min of incubation at room temperature, the reaction was measured at an absorbance of 415 nm. The total flavonoid contents were deducted through quercetin as a reference flavonoid compound. 

### 2.7. Total Soluble Protein and Carbohydrate Determination

The impact of Ag-NPs upon the total soluble protein (TSP) and total soluble carbohydrate (TSC) in the treatment plant groups under the CMV challenge were evaluated and compared to control plant groups as described in detail [34]. The TSP was evaluated according to the Bradford method [37] standard reference protein of bovine serum albumin. On the other side, total soluble carbohydrate (TSC) was evaluated according to [38] using the anthrone methods with a standard glucose curve. 

### 2.8. Impact of Ag-NPs on Antioxidant Enzyme Activity

#### 2.8.1. Polyphenol Oxidase (PPO) Activity Evaluation

The PPO enzyme was evaluated according to Quinone methods [39] as follows: 500 µL of plant tissue extract (phosphate buffer, pH 7.0) was mixed with 1 mL quinone solution. The quinone solution was prepared in 100 mM Tris-HCl buffer at pH 6.0 to a final concentration of 50 mM. After incubation for 10 min at 25 °C, the developing color was measured at 420 nm. The increase in the reaction absorbance by 0.001 represents one unit of the enzyme activity.

#### 2.8.2. Superoxide Dismutase (SOD)

The SOD activity was determined in all plant groups through photochemical reduction of nitroblue tetrazolium (NBT) [40]. First, the plant leaves samples (0.5 g) were homogenized in sodium phosphate buffer pH 7.0. The enzyme was evaluated by adding 100 µL of plant extract, 50 mM sodium carbonate, 0.1 mM EDTA, 10 µM riboflavin, and 12 mM L-methionine to a final reaction volume of 3 mL adjusted with phosphate buffer pH 7.6 (50 mM). The reaction mixture was exposed to a fluorescent lamp to initiate the reduction. After 15 min, the reaction was placed in the dark and measured at 560 nm. The reduction of the reaction color by 50% represents one unit of SOD activity (µmol/g of fresh weight). 

#### 2.8.3. Peroxidase Activity (POX)

The guaiacol reduction approach adapted from [41] was to evaluate the POX activity compared to control groups. The reaction included 80 µL of plant extract, 120 µL of 1 mM hydrogen peroxide, and 0.5 ML of guaiacol (5 Mm). The reaction mixture was adjusted to 1200 µL through phosphate buffer pH 7.0 (100 mM) and incubated at 30 °C for 10 min. The guaiacol reduction was measured at 480 nm, where results were deducted from the guaiacol extinction coefficient (ε = 26,600 M^−1^ cm^−1^).

### 2.9. Impact of Ag-NPs on Defense-Related Genes Expression and CMV Accumulation Level

The variation in gene expression levels in the squash plant under the CMV challenge was evaluated compared to control treatments through qRT-PCR. In this experiment, two polyphenolic controlling genes, including shikimate hydroxycinnamoyl transferase (*HCT*) and Chalcone isomerase (*CHI*) genes, in addition to three pathogenesis-related (PR) genes, including *PR-1*, *PR-2*, and *PR-5*, were also evaluated. Moreover, the relative transcriptional level of the CMV-coat protein gene (*CMV-CP*) was also investigated to check the accumulation level of the virus inside plant tissue. For the cDNA synthesis, the total RNA was extracted from all plant leaf samples according to the manufacturer’s instructions of RNeasy plant mini kit (Qiagen, Hilden, Germany). The total retrieved RNA was quantified through NanoDrop UV spectrophotometer (Labtech International Ltd., Sussex, UK) and used as a template for cDNA synthesis. The cDNA was synthesized using reverse transcriptase enzyme (Super-Script II, Invitrogen, Waltham, MA, USA), oligo (dT), and random hexamer primers with DNase I-treated RNA (2 μg) as a templet for each sample [42]. The qRT-PCR was performed using SYBR Green PCR Master Mix (Thermo Fisher, Carlsbad, CA, USA), and the amplified cDNA was applied as a template. The qRT-PCR reactions were run on Rotor-Gene 6000 (QIAGEN, ABI System, Germantown, MD, USA). The sequences of primers are listed in Table 1. To normalize the transcription level, the housekeeping gene, *EF1a,* was included as a reference in the qRT-PCR. According to the 2^−ΔΔCt^ algorithm [43], the relative transcriptional levels were accurately quantified and calculated. The transcriptional value >1 indicates gene accumulation (up-regulation) and values <1 indicate the opposite (down-regulation).

### 2.10. Statistical Analysis

GraphPad Prism software based upon analysis of variance (ANOVA) was used to evaluate the statistical significance of the analysis result at a probability value (*p*-Value) ≤ 0.05. The results’ significance was indicated in letters in descending order where (a > b > c) and the same letters indicated equal significance. All results were represented as means of triplicate with the corresponding standard deviations (SD) and error bars in histograms.

## 3. Results and Discussion

One of the most apparent signs that nanoparticles are being synthesized is a change in the color of the reaction mixture (seen visually) as time passes [44]. In the present investigation, the synthesis of Ag-NPs was indicated by the appearance of a reddish-brown color, which corresponds to the reduction of Ag+ to Ag° nanoparticles. Reducing silver ions to their corresponding NPs through plant extracts is widely reported as a practical, effective eco-friendly approach [45]. The reduction ability of *O. basilicum* leaves aqueous extract for several metals to nanometals was reported, including sulfur [46] and ZnO [47]. 

### 3.1. Morphological Characterization of the Biosynthesized Ag-NPs

**Figure 1:**
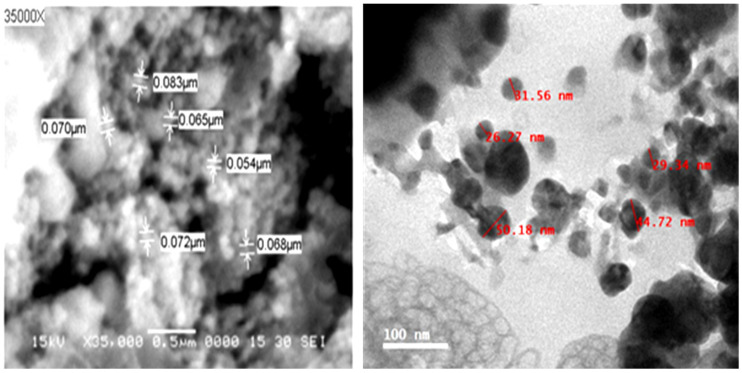
SEM (**left**) and TEM (**right**) images of silver nanoparticles biosynthesized by aqueous extract of *O. basilicum* as reducing agents. (Bar = 0.5 µm for SEM 100 nm for TEM).

The morphological structure of the prepared Ag-NPs was evaluated through SEM (Figure 1A). The results indicated varied round-shaped NPs, with different sizes ranging from 0.054–0.083 µm and an average particle size of about 0.069 ± 0.0086 µm (69 nm). The TEM results (Figure 1B) approved nanoscale Ag production in a range of 26.3–50.2 nm (average of 36.4 ± 9.3 nm) with spherical, hexagonal, and triangular shapes. The results agree with other studies that reported Ag-NPs in the same particle size through green synthesis with *Alhagi graecorum* and *Pistacia atlantica* extracts [27,48]. However, other Ag-NPs sizes were also reported, and the variation in Ag-NPs sizes and shapes could be attributed to the source of reducing agent and reduction conditions [49]. It was suggested that NPs with smaller particles have more surface area, boosting their biological activity by making it easier for them to penetrate biological membranes and target cellular structures [50]. As seen in Figure 1B, an organic layer may have formed around the NPs as a result of their interactions with the components of *O. basilicum* extract from leaves, which agrees with the Ag-NPs findings reported by Elbeshehy et al. [49]. This organic layer may be the cause of the particles’ resistance to aggregation and their increased biological activity [18].

### 3.2. Particle Size Distribution and Zeta Potential Analysis

**Figure 2:**
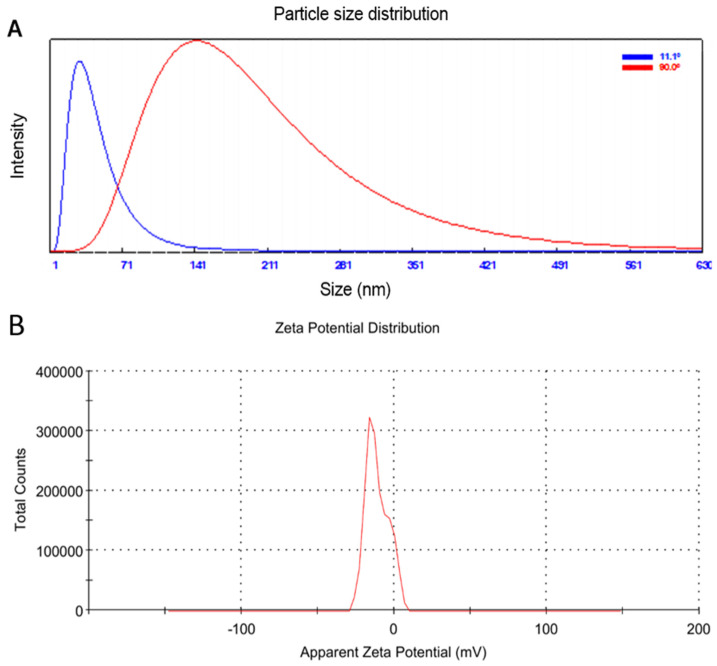
Particle size distribution using dynamic light scattering technique (**A**), and zeta-potential distribution analysis (**B**) of biosynthesized Ag-NPs.

A common technique is that particle size distribution in a colloidal solution can be determined with dynamic light scattering (DLS) [22]. The average particle size of the produced Ag-NPs in the aqueous medium was 32.6 nm at 11.1°, using DLS analysis in the current work (Figure 2A). The zeta potential indicated the surface charge power, which is directly related to the particle stability and toxicity [50]. As found in the literature, the zeta potential of around −30 mV characterizes particles with higher stability and lower toxicity to biological systems [51,52]. According to the results (Figure 2B), the generated Ag-NPs had good stability as evidenced by their low zeta potential of −11.1 mV [53,54]. The negative functional groups (COO-, OH-, and CO-) that were added to the surface of the produced Ag-NPs during their development from *O. basilicum* extract may be responsible for their net negative charge. Due to electrostatic repulsions, this negative charge promotes the stability of NPs [55].

### 3.3. EDS Analysis and FTIR Spectroscopy

**Figure 3:**
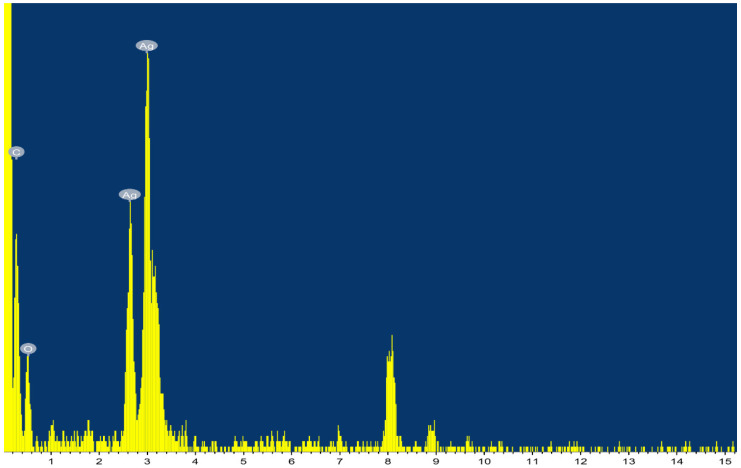
The EDX analysis of biosynthesized Ag-NPs using *Ocimum basilicum* leaf extract as reducing agents.

**Figure 4:**
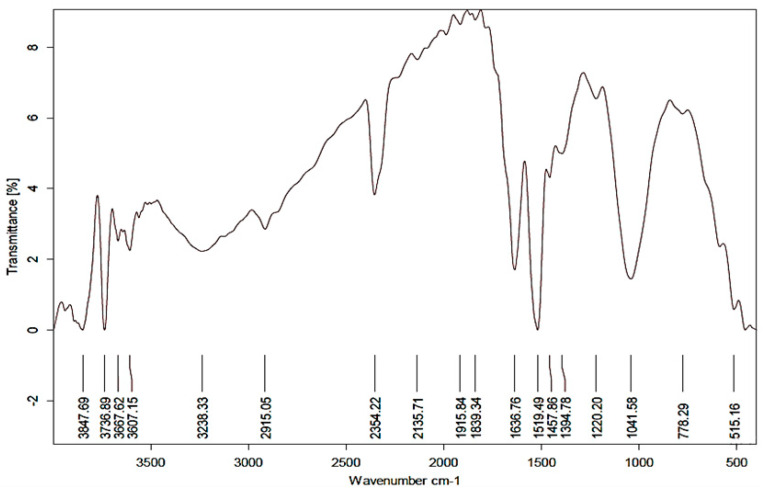
The Fourier transform infrared (FTIR) spectra of biosynthesized Ag-NPs using *Ocimum basilicum* leaf extract as reducing agent.

The EDX analysis approved the presence of a sharp peak at 3 KeV, related to metallic silver (Figure 3). The EDX spectrum shows the existence of silver nanoparticles as 80.69%. The presence of additional peaks (C and O_2_) was most likely caused by the glass that was holding the sample or could be attributed to the organic layer around the Ag-NPs [56,57]. On the other hand, the FTIR results (Figure 4) indicated a strong absorption band at 3736 cm^−1^, with a broad band at 3238 cm^−1^ usually attributed to the stretching vibrations of hydroxyl groups and primary amines [27,50]. The beak at 2915 cm^−1^ indicated the alkyl and CHO groups presence [58]. The strong band at 2354 cm^−1^, indicated C=O vibration of carboxylic groups, aldehydes, and ketones, similar to that reported in FTIR analysis of Ag-NPs prepared by different *Bacillus* sp. [49]. The band at 1636 cm^−1^ characterizes the vibration stretching for C=O carbonyl group [58], 1519 cm^−1^, and 1041 cm^−1^. Collectively, the FTIR results asserted the presence of organic capping of the prepared Ag-NPs of amide and carboxylic functional groups that accounted for Ag^++^ reduction, and sustained the stability of resulting NPs as previously reported [50,58].

### 3.4. Effect of Ag-NPs on Growth Parameters and Viral Accumulation Level

**Figure 5:**
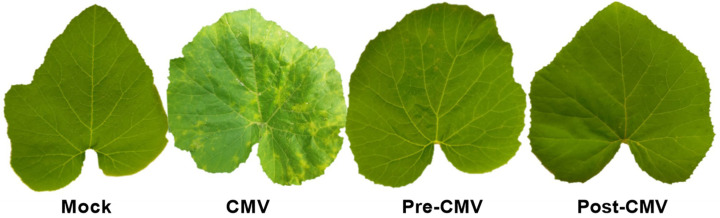
A photograph of disease symptoms on squash leaves infected with CMV 18 dpi. Mock: plants inoculated with viral inoculation buffer; CMV: plants mechanically inoculated with CMV; Pre-CMV: plants treated with Ag-NPs, 24 h before CMV inoculation; Post-CMV: plants treated with Ag-NPs, 24 h after CMV inoculation.

Under greenhouse conditions, squash plants that were mechanically inoculated with CMV (CMV treatment group) showed symptoms similar to CMV at 15 dpi. At 18 dpi, the symptoms were severe and looked like a mosaic (Figure 5). Severe chlorosis, yellowing, and mosaic symptoms, similar to those previously reported [9], are among the predicted signs. The findings are consistent with CMV’s potential to inhibit chloroplast production pathways via multiple methods [59]. The CMV-challenged groups, on the other hand, showed considerable morphological variation in both protective (pre-CMV) and curative (post-CMV) treatments, which could be related to the efficacy of Ag-NPs foliar spray to reduce CMV infection symptoms. The pre-CMV and post-CMV therapies, respectively, delayed the onset of symptoms by roughly five and three days.

The growth parameters measurements (fresh and dry weights) were in line with the morphological results described in Table 2. The results indicated a significant reduction in fresh and dry weights by about 25% in the CMV treatment group (7.29 and 0.72 g for fresh and dry weight, respectively) compared to mock-treatment plants (9.71 and 0.96 g for fresh and dry weight, respectively). The Ag-NPs foliar application alleviates the CMV effects on plant growth and weight. The pre-CMV treatment was slightly more effective than the application of Ag-NPs after infection (post-CMV) in enhancing plant growth (8.83 and 0.93 g for fresh and dry weight, respectively). Compared to CMV treatment, the pre-CMV treatment significantly increased fresh and dry weights by about 21 and 29%, respectively. On the other hand, the post-CMV treatment exhibited a considerable increase of about 15 and 22 % when compared to the fresh and dry weights of the CMV treatment, respectively. These results were similar to those indicating that silver nanoparticles increase plant development [17]. 

To relate and confirm the variation in the growth parameters to CMV infection, the relative expression level of *CMV-CP* was evaluated in all groups. The results (Table 2) indicated surge accumulation of *CMV-CP* in the CMV treatment group up to 83-fold compared to the Mock plants, which is in line with the mosaic symptoms and weight reduction results. Furthermore, the Ag-NPs foliar application significantly reduced the CMV-CP accumulation to 6.88 and 11.6 in pre-CMV and post-CMV treatment groups, respectively. The prophylactic Ag-NPs application was superior in retarding the viral accumulation representing a 12-fold reduction compared to the CMV group level. As intracellular pathogens, plant virus accumulation inside cells is an essential step in infection establishment and symptoms development; hence, the ability of Ag-NPs to retard viral accumulation could elucidate the enhancement in the morphological and growth parameters in the treated groups compared to the CMV treatment group. The results also asserted the importance of Ag-NPs application time, as prophylactic Ag-NPs application revealed significant results (regarding plant growth and viral accumulation) compared to treatment after 24 h of infection. These data provided support for the hypothesis that Ag-NPs are powerful antiviral factors. In this context, applying Ag-NPs to tomato plants reduced the disease severity and ToMV or PVY concentration levels inside plant tissues [32]. Furthermore, treatment with Ag-NPs after 24 h of virus inoculation lowered virus concentration and infection rate [60]. When Ag-NPs enter plant cells, they activate antiviral action (through DNA or RNA) and prevent viral reproduction by blocking the activity of cellular components or viral vectors [61]. Furthermore, Ag-NPs have been shown to bind to the viral genome, blocking polymerase activity and preventing virus replication [62].

### 3.5. Total Soluble Carbohydrate and Protein Determination

**Figure 6:**
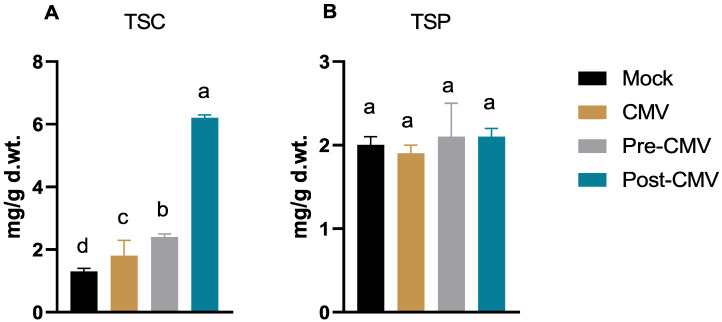
The impact of Ag-NPs foliar application upon (**A**) total soluble carbohydrate (TSC) and (**B**) total soluble protein (TSP) contents in squash plants. Mock: plants inoculated with viral inoculation buffer; CMV: plants mechanically inoculated with CMV; Pre-CMV: plants treated with Ag-NPs, 24 h before CMV inoculation; Post-CMV: plants treated with Ag-NPs, 24 h after CMV inoculation. Statistically, there is no variation between the means of the columns that share the same letter.

The results (Figure 6A) indicated a slight increase in the total soluble carbohydrate contents by about 38% in the CMV treatment group (1.8 ± 0.5 mg/g d. wt) compared to mock-treatment plants (1.3 ± 0.1 mg/g d. wt). In contrast, many studies have found that plant viral infection reduces the total soluble carbohydrate content [34,63,64]. The findings are consistent with those of Gonçalves et al. [65], who discovered that the sugarcane yellow leaf virus (ScYLV) enhanced the total soluble glucose content of sugarcane. Foliar application of Ag-NPs enhanced the total carbohydrate contents (2.4 ± 0.1 mg/g d. wt) in pre-CMV treatment, while in post-CMV, the carbohydrate contents significantly surged to 6.2 ± 0.1 mg/g d. wt, representing about a 4.8-fold increase compared to the Mock group (Figure 6A). On the other hand, no significant change in the total soluble protein content was reported among different treatments (Figure 6B). 

### 3.6. Evaluation of the Free Radical Scavenging Activity, Total Phenolic, and Flavonoid Contents

**Figure 7:**
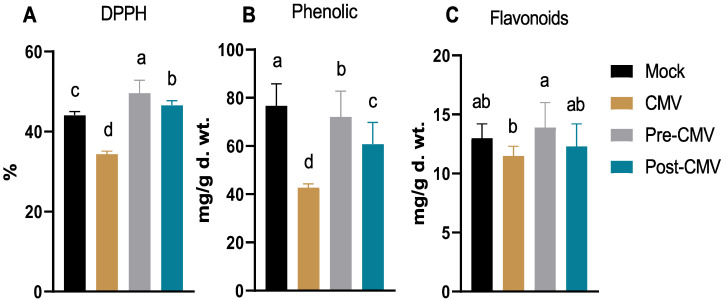
The impact of Ag-NPs foliar application on free radical scavenging activity using DPPH (**A**), total phenolic (**B**), and flavonoid contents (**C**) in squash plants. Mock: plants inoculated with viral inoculation buffer; CMV: plants mechanically inoculated with CMV; Pre-CMV: plants treated with Ag-NPs, 24 h before CMV inoculation; Post-CMV: plants treated with Ag-NPs, 24 h after CMV inoculation. Statistically, there is no variation between the means of the columns that share the same letter.

As indicated in Figure 7A, the free radical scavenging activity was reduced in the CMV treatment group (34.4%) by about 22% compared to the activity in Mock plants (44.1%), which may be related to the squash’s defense against viral infection to mitigate the negative effects of the surge in oxidative stress [66]. The treatment with Ag-NPs enhanced the free radical scavenging activity to 46.6% in the post-CMV group, with maximum enhancement in the pre-CMV treatment to about 49.6%. The maximum free radical scavenging in pre-CMV treatment represents about 12- and 44% increases compared to Mock and CMV treatments. Such results indicated that the antioxidant capacity of the biosynthesized Ag-NPs using plant extract showed relatively high scavenging activity [67,68,69,70].

It is common knowledge that plant polyphenolic chemicals, such as phenolic and flavonoid compounds, play a significant role in the plant’s ability to defend itself against biotic and abiotic stresses, such as viral infections [23,71,72]. The obtained results indicated a reduction in both phenolic and flavonoid contents (42.8 ± 1.5 and 11.5 ± 0.8 mg/g d. wt, respectively) in CMV treatment compared to Mock plants (76.7 ± 9.1 and 13 ± 1.2 mg/g d. wt, respectively) which is in line with the free radical scavenging activity (Figure 7B,C). The reduction of phenolic and flavonoid contents was reported among the main traits for several viral plant infections [73,74] and could be attributed to viral suppressor activity and severe mosaic symptoms [75]. The treatment with Ag-NPs revealed parallel impacts on phenolic and flavonoid contents, where the pre-CMV treatment significantly enhanced both contents to 72.1 ± 9.7 and 13.9 ± 2.1 mg/g d. wt. Although both phenolic and flavonoid levels increased by roughly 68 and 20 percent in pre-CMV treatment compared to the CMV group, the highest phenolic and flavonoid contents in the pre-CMV treatment were approximately in the same ranges as Mock plant groups. The results are corroborated by earlier studies [76,77].

### 3.7. Impact of Ag-NPs on Antioxidant Enzymes Activity under CMV Challenge

**Figure 8:**
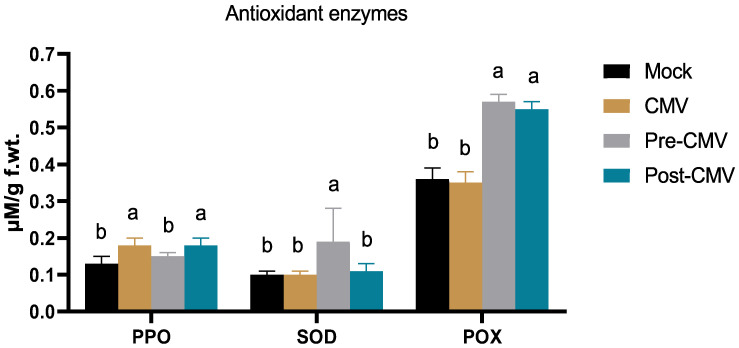
The impact of Ag-NPs foliar application on three antioxidant enzymes (PPO, SOD, and POX) in squash plants. Mock: plants inoculated with viral inoculation buffer; CMV: plants mechanically inoculated with CMV; Pre-CMV: plants treated with Ag-NPs, 24 h before CMV inoculation; Post-CMV: plants treated with Ag-NPs, 24 h after CMV inoculation. Statistically, there is no variation between the means of the columns that share the same letter.

Plant viral infections are characterized by the over- and un-controlled expression of reactive oxygen species, which causes oxidative stress in the cell and may interfere with many vital plant processes [78,79]. As shown in Figure 8, the PPO enzyme was enhanced by about 38% in the CMV treatment (0.18 ± 0.02 µM/g f. wt) compared to the Mock plant group (0.13 ± 0.02 µM/g f. wt), which could be attributed to the initial squash response to the viral infection, which is in line with ZYMV infection in the same plant [34]. In the same regard, the Ag-NPs enhanced the enzyme activity to 0.15 ± 0.01 and 0.18 ± 0.02 µM/g f. wt for pre-CMV and post-CMV treatments, respectively. The PPO plays a significant role in lignin deposition as a physical barrier against many phytopathogens [34]. Lignin mediated through PPO resulted in an end product for scavenging many reactive oxygen species through phenolic compounds, hence its antioxidant activity [80]. The SOD enzyme mediated the initial neutralization of the highly reactive free superoxide species (O_2_^−^) into hydrogen peroxide (H_2_O_2_) that subsequently detoxified through several plant enzymes (as catalases and glutathione peroxidases) into O_2_ and water [74]. The results show no significant difference in SOD activity among CMV and post-CMV treatment compared to mock-treatment plants (Figure 8). Upon Ag-NPs application before viral infection, the SOD level was significantly enhanced up to 0.19 ± 0.09 µM/g f. wt in pre-CMV (Figure 8). Thus the pre-application of Ag-NPs could trigger the induced systemic resistance to viral infections through the secretion of enzymes involved in the immune response [81]. In the same regard, the POX is an essential antioxidant enzyme with several reported defensive mechanisms within plant cells. Some POX classes (class III) are directly involved in lignin formation and cell wall enforcement toward different phytopathogens [82]. In addition to entry and movement retardation mechanisms, the enzyme interferes with plant viral replication and enhances plant resistance [83,84]. As shown in Figure 8, the foliar application of Ag-NPs significantly enhanced the POX production to 0.57 ± 0.02 and 0.55 ± 0.02 in pre-CMV and post-CMV treatments, respectively, compared to Mock plants (0.36 ± 0.03 µM/g f. wt). The maximum POX enhancement in the pre-CMV group represented about a 63% increase in the enzyme titer compared to the CMV group, indicating the importance and direct involvement of the POX enzyme in squash resistance to the CMV challenge. As a result, the Ag-NPs might activate such enzymes to stop CMV from spreading and multiplying inside squash plant cells by creating polymerized phenolic barriers surrounding infection sites to eradicate the virus [85,86]. 

### 3.8. The Impact of Ag-NPs Application on Gene Expression under CMV Challenge 

**Figure 9:**
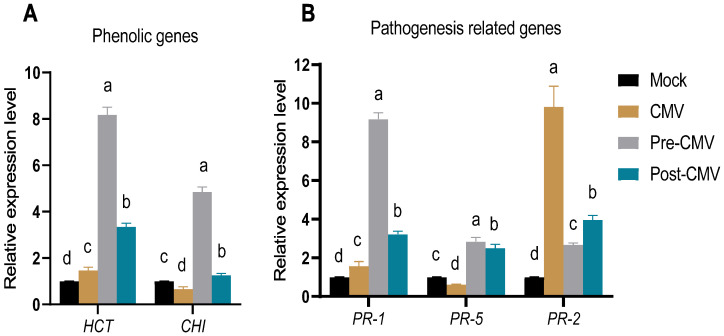
The impact of Ag-NPs foliar application on five gene expressions in squash plants. Mock: plants inoculated with viral inoculation buffer; CMV: plants mechanically inoculated with CMV; Pre-CMV: plants treated with Ag-NPs, 24 h before CMV inoculation; Post-CMV: plants treated with Ag-NPs, 24 h after CMV inoculation. Statistically, there is no variation between the means of the columns that share the same letter.

The effect of Ag-NPs foliar application on the expression of polyphenolic and pathogeneses genes of squash plants under the CMV challenge was evaluated in all treatment groups. Shikimate hydroxycinnamoyl transferase (*HCT*) is an essential enzyme for the biosynthesis of lignin precursors and hence enhances the plant’s defense by enforcing the plant cell wall [87]. As depicted in Figure 9A, the *HCT* activity was slightly enhanced in the CMV treatment group by 47% compared to Mock plants, which could be attributed to the induction of plant defense against viral infection [88]. The treatment with Ag-NPs surged the *HCT* accumulation to 8.2- and 5.6-fold increases compared to mock treatment. As a result, the increase in *HCT* transcriptional expression reveals its curative and protective effects against CMV and raises the possibility that the squash plant may be able to use lignification as a line of defence against viral infection and dissemination in response to foliar Ag-NP treatment. In the same vein, the Ag-NPs treatment plant examined *CHI*, one of the crucial enzymes in the flavonoid production pathway (Figure 9A). In contrast to mock plants, the CMV treatment plants showed a 33 % lower relative expression level, indicating that CMV infection may inhibit naringenin production [87,88]. The foliar application of Ag-NPs (pre-CMV and post-CMV) intriguingly revealed high levels of *CHI* that are strictly required for flavonoid production in different plant tissues [89,90]. Meanwhile, Ag-NPs treatment might be an efficient elicitor for the induction and accumulation of flavonoids [91]. 

Furthermore, three PR genes were evaluated in all treatment groups. The *PR-1* is a marker for salicylic acid (SA) accumulation which is the primary mediator for systemic acquired resistance (SAR) regulation [92]. Thus it can directly regulate the SAR against many biotic and abiotic stresses [34]. In the current study, *PR-1* was slightly activated in the CMV treatment group, about 57% higher than in non-challenged Mock plants (Figure 9B). Application of Ag-NPs significantly increased the *PR-1* transcript with relative expression levels of 9.2- and 3.2-fold in pre-CMV and post-CMV treatments, respectively, higher than in the Mock group (Figure 9B). The *PR-5* is a critical defensive gene; it is a thaumatin-like protein widely upregulated toward several phytopathogens [93]. When compared to the Mock plants, PR-5 evaluation results demonstrated a considerable downregulation in expression level in CMV treatment plants by about 39%, which might be attributed to viral suppressor activity. Foliar application of Ag-NPs significantly increased *PR-5* transcript levels, with 2.8- and 2.5-fold increases in pre-CMV and post-CMV treatments, respectively, when compared to the Mock plants. Therefore, foliar spraying of squash plants with biologically produced Ag-NPs, either before or after inoculation with CMV, induced the expression of *PR-1* and *PR-5* genes, which may result in increased SA accumulation [94]. Based on these results, it seems that the biosynthesized Ag-NPs made by *O. basilicum* may trigger induced systemic resistance (ISR) and play a vital role in activating the SAR pathway in squash plants against CMV infection. The *PR-2* gene encoded the β-1,3-glucanase that was reported to facilitate the intracellular cell-to-cell movement of plant viruses through its callose-hydrolyzing activity [95,96]. Furthermore, when CMV was applied to plants, the *PR-2* gene was dramatically elevated, up to a 9.8-fold increase as compared to the Mock plants. As a result, overexpression of *PR-2* increased the severity of the viral infection, which is consistent with earlier observations of a clear upregulation of *PR-2* during viral infections in diverse plant species [34,71,97,98,99]. This suggests that Ag-NPs applied before or after viral infection may mitigate CMV infection by suppressing *PR-2* expression and blocking intercellular spread across long distances. 

## 4. Conclusions

The current findings indicate the potency of biosynthesized Ag-NPs mediated by aqueous extract of *O. basilicum* against the cucumber mosaic virus. DLS, SEM, and TEM analyses revealed that the Ag-NPs were spherical, ranging from 26.3 to 83 nm, with an average particle size of about 32.6 nm. Greenhouse experimental results revealed that the foliar treatment of squash plants with Ag-NPs (100 µg/L) induced SAR, decreased disease severity, and reduced CMV accumulation levels by up to 92%. Additionally, it promoted growth parameters and induced the expression levels of *PR-1*, *PR-5*, *HCT*, and *CHI* genes. It increased total soluble carbohydrates, DPPH, PPO, SOD, and POX, and total phenolic and flavonoid content. For this reason, employing Ag-NPs as a potential inducer for systemic resistance in squash against CMV infections may be a useful alternative technique for dealing with plant viral diseases without resorting to pesticides. Nonetheless, more investigation is needed to confirm these findings in the open field and to comprehend the underlying mechanisms.

There is still a lack of knowledge on how AgNO_3_ (the precursor of Ag-NPs) affects numerous plants, including *Cucurbitaceae*. As a result, the findings of the following study could be applied to toxicological studies, leading to the discovery of methods for mitigating AgNPs’ and AgNO_3_’s deleterious effects on agricultural plants.

## Figures and Tables

**Table 1:** Nucleotide sequences of primers used in this study.

Primer Name	Abbreviation	Direction	Nucleotide Sequence
Cucumber mosaic virus-coat protein	*CMV-CP*	Forward	GGATGCTTCTCCACGAG
		Reverse	AGTGACTTCAGGCAGT
Hydroxycinnamoyl transferase	*HCT*	Forward	TCTCCAACCCCT TTTAACGAACC
		Reverse	CAACTTGTCCTTCTACCACAGGGAA
Pathogenesis related protein-1	*PR-1*	Forward	CCAAGACTATCTTGCGGTTC
		Reverse	GAACCTAAGCCACGATACCA
Chalcone isomerase	*CHI*	Forward	GGCAGGCCATTGAAAAGTTCC
		Reverse	CTAATCGTCAATGATCCAAGCGG
Endoglucanase	*PR-2*	Forward	TCAATTATCAAAACTTGTTC
		Reverse	AACCGGTCTCGGATACAAC
Thaumatin-like protein	*PR-5*	Forward	CCGAGGTAATTGTGAGACTGGAG
		Reverse	CCTGATTGGGTTGATTAAGTGCA
Elongation factor 1-alpha	*EF1a*	Forward	ATTCGAGAAGGAAGCTGCTG
		Reverse	TTGGTGGTCTAAACTTCCAC

**Table 2:** The growth parameters of squash plants under CMV challenges with corresponding viral accumulation levels as affected with Ag-NPs foliar treatment.

Treatment Groups	Growth Parameters		The Relative Expression Level of *CMV-CP*
	Fresh Weight	Dry Weight	
Mock	9.71 ± 1.21 a	0.96 ± 0.12 a	00.00 ± 0.01 d
CMV	7.29 ± 1.02 d	0.72 ± 0.22 d	82.71 ± 1.94 a
Pre-CMV	8.83 ± 0.92 b	0.93 ± 0.23 b	06.88 ± 0.94 c
Post-CMV	8.37 ± 1.02 c	0.88 ± 0.25 c	11.66 ± 0.98 b

Mock: plants inoculated with viral inoculation buffer; CMV: plants mechanically inoculated with CMV; Pre-CMV: plants treated with Ag-NPs, 24 h before CMV inoculation; Post-CMV: plants treated with Ag-NPs, 24 h after CMV inoculation. Statistically, there is no variation between the means of the columns that share the same letter.

## Data Availability

Not applicable.
